# The politics of suspension suspended: the curious case of a cryopreserved cell product

**DOI:** 10.1057/s41292-024-00328-z

**Published:** 2024-07-09

**Authors:** Ruzana Liburkina

**Affiliations:** Faculty of Social Sciences, Goethe University Frankfurt, Theodor-W.-Adorno-Platz 6, 60323 Frankfurt am Main, Germany

**Keywords:** Suspension, Politics, Cryopreservation, Innovation, Preparedness, Donation

## Abstract

Following recent discussions around suspended life, this paper focuses on an endeavor that sought to arrest biological material in time and space and render it available on demand. It depicts the attempt to establish a collection of cryopreserved donated cells. The study offers rare insights into how this initiative was at odds with familiar politics significant in its field, those of innovation and preparedness, and therefore was suspended itself. In identifying parallels with accounts of unsuccessful biobanks, the paper makes a case for the analytical value of considering ill-fated projects of suspension along with those that prosper and attract public attention. The case of a novel cryo-collection, in particular, demonstrates how the idea and practice of suspension only gathers political momentum when it serves other well-established rationales. As such, it prompts two important conclusions. First, the power to arrest life as it comes with cryotechnologies is much more likely to unravel in entrenched constellations than to carry transformative or disruptive potential. Second, however, the paper also exemplifies that projects of suspension are not necessarily doomed to serve hegemonic ways of governing life. It advocates for preventing such mismatches from falling into oblivion.

## Introduction

Manipulating life forms by pausing their decay is an “unprecedented power over life” (Friedrich and Hubig 2018, p. 173, author’s translation) that deserves explicit attention: this is the basic and apt assumption shared by the now considerable community of scholars who have undertaken the study of cryopreservation (e.g., Radin and Kowal 2017a; Kroløkke et al 2020; Katz et al 2020; Braun et al 2023). This paper demonstrates how this particular form of power is not only embedded in but also highly conditional on others. It does so by drawing on the recent discussion of the power to arrest life as a manifestation and result of a politics of suspension (Lemke 2023b). In shifting the focus from the hitherto prevalent term “cryopolitics” (Radin and Kowal 2017a) to the notion of suspension, Thomas Lemke has put a stronger emphasis on the ideals of reversibility and availability as driving forces for cryobanking projects. Beyond “not letting die” (see also Radin and Kowal 2017b, p. 8; Friedrich and Höhne 2014), Lemke argues, cryotechnologies are also used to defer looming loss and unpleasant futures. As such, they bear great political potential: they facilitate a new way of governing life and time in the name of particular social norms and objectives. Lemke observes attempts to pursue this by arresting biological material in a liminal state of being neither alive nor dead and rendering it available on demand (Lemke 2019, 2023a). As the empirical case depicted here shows, this is indeed an appealing logic that is enthusiastically embraced by a range of different actors. However, it also indicates that notwithstanding its instigating nature, the idea and practice of suspension only gathers political momentum when it serves other well-established rationales.

The idea of making living cells available as a means of facing the risk of irreversible loss was precisely the one lurking behind the project I studied. The project set out to complement an existing donation system by a pool of cryopreserved products. Regardless of its noble motives and compliance with what Lemke (2023b, p. 713, see also 2023a) characterizes as a proliferating “mode of future-making” and managing life, however, the implementation of this project was defined by various unexpected obstacles, throwbacks, and delays. Thinking through these rare insights into a case of a pending, not yet finalized, project of suspension prompts important conclusions on the characteristics of the corresponding politics that have been identified. Cryotechnological suspension, I shall argue, does not necessarily gain political traction—either in specific terms of biopolitics (Lemke 2011, 2019) or in terms of politics as a site of potential negotiation, dispute, and rearrangement more broadly. It is not always an impactful vehicle of governing lives, and it may well not make any difference at all in either promoting or subverting ideologies. The idea of suspension, it turns out, only becomes political under particular conditions, namely as an extension of established political forms. Otherwise, it is itself suspended in favor of more commensurable ones.

After introducing the case of the aforementioned cell product, I will draw attention to empirical social studies of biobanking that describe difficulties in attempts to align to various political rationales. I shall argue that these findings are symptomatic of the conditional nature of the politics of suspension in general. The latter have hitherto been exclusively discussed by taking the example of projects that are deduced from and meant to reproduce other politics and power formations. Yet, some projects of suspension may well be organized in unaccustomed ways that fall through the cracks of established political conjunctions. Suspending life, *then*, does not unfold as a form of “unprecedented power”; it remains stuck in its status of an appealing idea, a mere intention, with no politics gathered around it. My research on such a case interrogates generalizations of the political force of cryotechnological suspension. This includes both effusive enthusiasm on the part of life science practitioners and criticism from social scientists. The practice of suspending life does not hold far-reaching power per se but gains it through an alignment with dominant political logics.

In putting forward this hypothesis, I dedicate the empirical part of this paper to insights into how the cryopreservation of a particular cell product as an envisaged project of suspension failed to resonate with the beaten tracks of politics relevant for its field: (1) the prioritization of innovation and research economies in regulating biomedical research and development and (2) rationales of preparedness in the context of the COVID-19 pandemic. To better understand the dynamics and implications of falling through the cracks of politics, I draw on the notion of “outside politics” coined by Niamh Stephenson and Dimitris Papadopoulos (2006a, 2006b). As a heuristic device, this notion renders tangible how a project of suspension that is incommensurable with familiar political rationales does not bear political potential. The paper ends with concluding remarks on what we can learn from the case of a project of suspension as a mismatch and on the potential epistemic advantages of reconsidering the politics of suspension as conditional.

## Case study

This paper is based on my work in a European research project on the cryopreservation of human and non-human biological material (CRYOSOCIETIES). To develop a differentiated STS perspective on the phenomenon of suspended life, my colleagues, Thomas Lemke, Veit Braun, Sara Lafuente-Funes and I are conducting research in and around several different research and clinical biobanks around Europe. These are organizations specialized in preserving, storing, administering, and releasing specimens. Our research activities include ethnographic fieldwork, semi-structured interviews, online surveys, as well as media, document and policy analysis. It was through this heterogeneous empirical work that I became acquainted with the case presented in this paper. As my inquiry depicts ongoing project development work and revolves around an organization that, as a matter of fact, is expected to attune to politics in order to survive, I must remain sensitive to the possibility that my argument could have a negative impact on how it is perceived. To ensure the anonymity of the actors involved, I abstain from providing any information that might render them identifiable. I leave entirely open in which country they were located, what types of biobanking activities they pursued, and what kinds of research material I collected. For the same reason, I will not specify the exact type of cells at the center of the project that gave rise and form to the argument presented here. Nevertheless, I make sure to provide all information necessary for making sense of both the main prerequisites and characteristics of the project and the obstacles and challenges it encountered.

Cell banking is a vast field that includes myriad different organizations, infrastructures, and products. As such, it can hardly be adequately presented as a whole in a methods section of a social-scientific paper. The project of suspension to be discussed in this paper revolves around a particular kind of biological material that up until then had rarely been systematically cryopreserved. As is the case with most cell types considered valuable for therapeutic or reproductive purposes, these cells—let me call them A-cells—can be extracted from and donated by one person and transplanted to another. There are several types of cells that currently circulate in such cellular gift economies: egg cells, sperm cells, white blood cells, hematopoietic stem cells, red blood cells, and thrombocytes. Many of these cells can be obtained from several different tissues and substances, including blood, bone marrow, the umbilical cord, placenta, adipose tissue, dental pulp, immortalized cell lines, engineered cells, and others.

One of these cell types, namely cord blood stem cells, can only be made available through cryopreservation. Another one, sperm cells, is almost exclusively cryopreserved; fresh sperm donations mainly take place in non-institutional settings. Finally, egg cells have routinely been cryopreserved since the 2010s, with the rate of fresh donations still higher than that of ‘frozen’ ones but rapidly decreasing (Tober et al 2023). The donation of the remaining cell types, including A-cells, is normally not mediated by long-term and low-temperature storage facilities. Instead, it relies on what Violeta Argudo-Portal (2023) has called “walking biobanks”—the bodies of donors who can be approached and asked to provide their cells.

There are two possible ways of accessing “walking biobanks,” depending on the probability of finding a match between a donor and patient. The gift economy of blood stem cells demands a high histocompatibility, which is rare and difficult to find. Therefore, it is ordered around transnational registries of potentially available donors who are contacted once they are identified as a match. The donation system of whole blood components such as red or white blood cells, in turn, requires a compatibility of blood types. As the latter is normally much more probable than histocompatibility, the gift economy of blood components is mainly grounded on spontaneous and on-demand donations. Here, “walking biobanks” are expected to show up or encouraged to do so in public campaigns. However, there are patients whose blood types have rare antigens or lack common ones—finding a donor is then as difficult as in the case of stem cells. Therefore, the gift economy of blood components also partly operates through registries. These registries contain data of so-called “target donors who are known to have […] requested rare phenotype[s]” (Laureano et al 2023).

What seem like self-evident procedures are inherently fragile systems. The COVID-19 pandemic made this particularly evident by quickly disrupting the entire gift economy of cell donations. As this economy mainly includes short-term storage infrastructures and relies on time-efficient logistics and a rapid mobilization of people and biological material, the unexpected obstructions to free movement as they occurred in the early stage of the pandemic posed a serious threat to its functionality. Suddenly, the availability of “walking biobanks” was limited as neither people nor freight could necessarily move as envisaged and required for a quick transplantation of fresh biological material. Many potential donors were either afraid to travel to a donation center or simply could not do so because of “government’s interventions such as home sheltering, mass lockdown, and curtailment strategies” (Sahu et al 2020, p. 105). In the case of blood stem cell donations, some of the already requested donors turned out to be in quarantine or even “contracted COVID-19 after the recipient started conditioning chemotherapy” (Maurer et al 2022). The pandemic also “affected blood supply around the world” (Halawani 2022, p. 3) and consequently caused significant shortages of blood components.

This is where my case study comes in—a case of entrepreneurial spirit, a group of people who enthusiastically embraced the ideas that underpin what Lemke calls a politics of suspension. These were the people in charge of running the biobank I am calling X-Bank. Even before COVID-19, its managing directors (partly physicians themselves) set out to use their expertise and infrastructural capacities to fix the vulnerability of the system of cell donations by making A-cells—typically enrolled in short-term donation—persist in time. The main idea behind the project was to do away with the short time frames of their shelf life and the irreversible temporalities of donation and transportation, and to allow the entire process to be more flexible. It was designed to make A-cells available for yet-unknown future needs and emergencies through a collection of units that would be stored in a cryobank and be available for shipping and transplantation whenever and wherever needed.

As a stockpiling project, this cryo-collection was supposed to complement the system of donation and transplantation of fresh cells and “to act as a buffer against [its] disruptions” (Folkers 2019, p. 495).^1^ Cryopreserved A-cells (cryo-A-cells) are meant to stay in place and to persist in time, unaffected by any logistical difficulties, health crises, or delays. Once put in a liquid nitrogen tank shortly after donation, the units are safely contained and basically immune to crisis events.^2^ They are envisaged as an additional “standing reserve” (Lemke 2023b; Argudo-Portal 2023) of A-cells that can be accessed notwithstanding the availability of freshly donated material. As such, X-Bank’s initiative is a matter of risk mitigation: its installment promises a differentiated gift economy of cells that is more resilient if difficulties arise. What seems to be a largely uncontroversial idea, however, proved to be very hard to implement. When I submitted this paper several years after X-Bank called the project into life, it was still in the projection phase.

I did not intend to study the projection and organization of a project of suspension, but came to do so during my research on the established landscape of cell banking. In other words, I was expecting to study cryotechnological suspension as routine and yet ultimately also became interested in it as a project in the making. The following description and analysis of X-Bank’s cryo-A-cells project and its struggles resulted from a process of coding, summarizing, ordering, and re-visiting more than 300 typed pages of diverse empirical material against the backdrop of different theoretical debates (Emerson et al 2011). Engaging with literature on cryotechnologies, research governance, biobanks, preparedness, and the margins of politics enabled me to make sense of these surprising findings in a nuanced manner (Timmermans and Tavory 2012). In line with Malte Ziewitz’s suggestion, I “expose[d my] own assumptions and put them up for grabs” (Ziewitz and Lynch 2018, p. 382).

## Beyond success stories: suspension viewed from its ill-fated end

The cryo-A-cells project was a clinical biobanking initiative, which means that the biological material itself was meant to be made available for therapeutic rather than research purposes. Therefore, and in light of its exclusive focus on ultralow-temperature preservation, I call it a *cryo*banking endeavor. After all, the term ‘biobank’ is mainly used to name a facility that preserves specimens to be accessed by researchers. Despite their obvious differences in terms of relevant networks, norms, and procedures, both clinical and research biobanks engage in suspending (mainly molecular and cellular) life forms on demand. The latter, however, tend to gain more attention as specific organizations operating in complex institutional contexts. Thus, there are several science and technology studies (STS) accounts of research biobanking projects failing to find their place in the political organization of biomedicine and the life sciences. In developing a typology of biobanks in the early 2010s in the US, Robert Mitchell (2012) first depicted how the strategies of some institutions fell through the cracks of political agendas and regulation by either making too many incompatible promises or not making any politically effective ones. Neil Stephens and Rebecca Dimond (2015), in turn, traced the unmet expectations that led to the closure of a biobank. Their case study demonstrates how this particular biobank’s position outside academic and healthcare networks resulted in its activities being considered “a distraction” (ibid., p. 426) no one actually benefitted from. In a similar vein, Erik Aarden’s (2017) account of the closed Singapore Tissue Network illustrates how biobanking projects need to align to the micropolitics of research if they are to be used and promoted. Most recently, drawing on Stephens and Dimond’s as well as on Aarden’s work, Violeta Argudo-Portal and Miquel Domènech (2022, p. 330) have identified a general “precariousness of biobanks” as public service providers and storage facilities. Their empirical study of attempts of Spanish biobanks to counteract this unfortunate position renders visible how these organizations increasingly reject the logics of the politics of suspension in favor of immediate usefulness. Instead of operating as stockpiling facilities for future needs and opportunities, they turn to “encouraging project-based collection of samples and data” (ibid., p. 341) the utilization of which is certain and predictable.^3^ This contradicts the idea and ideal of rendering biological material available at any point in time, which Lemke (2023b, p. 705) depicted as “the principle of ‘whenever’”—the linchpin of the governance of life by means of cryopreservation. The principle of whenever implies that life forms are preserved in anticipation of future situations and conditions in and under which they might be needed and utilized. In the case of Spanish biobanks, however, the imperative to suspend is radically reversed to “staying with the present, rather than relying on postponed actions and promises” (Argudo-Portal and Domènech, 222, 341).

The aforementioned case studies focus on the field of research biobanking and the specific regulatory, social, and epistemic challenges that come with it. They also draw attention to another, broader issue: preserving biological material so that it will be available at any time does not always come to make a difference. It does not always fulfill its envisaged political potential and become a vehicle of governing life, time, and knowledge production. Even when projects of suspension are initially framed as infrastructural fixes, they can fail to sustain this promising status. Such organizations either close down or dismiss the rationale of suspension if they fail to perform as an extended arm of the politics that already order the field in which they operate.

In introducing the notion of a politics of suspension, Lemke (2023a, b) identifies three fields that illustrate its significance and logic: seed banking, de-extinction initiatives, and non-medical egg freezing. He observes that in all three domains, cryotechnologies are mobilized to detach life “from the network of biological, ecological, and social interactions it originated from” (Lemke 2023b, p. 710) and to extend the time frame for biotechnological interventions. While this mode of managing life is certainly “distinctive” (ibid.), I argue that it is important to pay explicit attention to how well it blends in with and responds to the political rationales already in place and at play, respectively. Suspending agricultural seeds, for instance, fits perfectly into the logics of the politics of security (Collier and Lakoff 2021) and agribiopolitics (Hetherington 2020) as they unfold in the governance of industrialized agriculture (Wolff 2021). With its vertical institutional structure, this practice also resembles established power ecologies in this field (Henke 2008; van Dooren 2007). Similarly, rather than manifestations of abstract ideas of suspension, de-extinction science projects are first and foremost situated in the midst of venture capital and represented by public figures such as George Church, who are well-known for cherishing and pursuing transhumanist and eco-modernist visions (Business Wire 2023). As such they are consequential in facilitating techno-utopian agendas. So-called social freezing, in turn, is not an instance of a distinct kind of politics but a paradigmatic case of a commercial (and financial) appropriation of a clinical practice and thus an instance of capitalization (Muniesa et al 2017; van de Wiel 2020). Besides, it is exemplary for neoliberal ideas of how biotechnologies should facilitate ways of self-government that are compatible with economic uncertainties and market rationalities (Baldwin 2018; Carroll and Kroløkke 2018).

In all three of these domains, isolating life forms to render them available for interventions on (future) demand is a means to predefined ends. In all three cases, it comes as a technological solution to political problems long posed as such—be it by the politics of security, eco-modernist imaginaries, or the logics of commercialization and responsibilization. However, *not all* projects of biotechnological suspension are necessarily projects that render “the future […] increasingly trapped by present choice” (Strathern 1995, p. 61). The power to arrest life is not always mobilized to sustain and perpetuate familiar discourses, social orders, imaginaries, and ways of governing. Studies of unsuccessful research biobanks document attempts to offer solutions for problems yet to be framed and defined. The cryo-A-cells project depicted in this paper also shows that initiatives to suspend biological material may well fail to fit in. What is more, it even illustrates that they can be called into life from beyond well-known “aggregate[s] of governance” (Stephenson and Papadopoulos 2006a, p. 154). In what follows, I will ground this claim in a differentiated account of how the ideas and practices around cryo-A-cells struggled to align with other politics.

## Suspension at odds with research and innovation politics

First and foremost, the cryo-A-cells project as a project of suspension was not attuned to research and innovation politics. Of course, the cryopreservation of A-cells is ultimately a process of industrialization: what used to be an on-demand gift economy is complemented by a laborious manufacturing procedure. As such, this endeavor comes with a technological apparatus, specialized production facilities, and skilled labor. However, as a cryobanking initiative meant to serve clinical purposes only, it does not bring with it a research economy. There was no one beyond X-Bank’s organizational boundaries to take economic advantage of its establishment—neither in terms of immediate profit, nor in terms of social and cultural capital, regional development, scientific infrastructure, an expansion of the biotech sector, the advancement of global or national health information markets (Wyatt et al 2020; Tupasela 2021), or anything like this. Neither the state as an economic actor in global biotech economies nor the particular region where the envisioned cryo-collection was supposed to be located were likely to benefit from this project. There was no promise of “enhanced growth […] and productivity” (Morrison 2017, p. 76), and “the future work that […] will be carried out using the material” (ibid., p. 67) does not have calculable economic value. Hence, the other side of the coin of its generally uncontroversial nature is the fact that there are no major high-stakes expectations that are addressed by the cryo-A-cells project. However, it is expectations that result in investment—be it in terms of funding or regulatory support (Borup et al 2006).

By falling through the cracks of research and innovation politics, the cryo-A-cells project could not thrive in and benefit from a regulatory context that Alex Faulkner (2009) has described as “governation”—a systematic coupling of the apparatuses and logic of governance and innovation. This coupling is particularly important for any kind of bioeconomies, as they are framed and ordered by the imperative and the rhetoric of innovation (Braun 2021). Drug and healthcare regulation in particular tends to be organized along the poles of innovation and control (Engel 2012), with the former being defined by a “move toward acceleration” (Webster 2019, p. 1) and the latter by very limited flexibility. In failing to be considered as an instance of research innovation, the cryo-A-cells project was fully exposed to the rigid, control side. It was subject to something that can be characterized as *bare* regulation: regulation free of interest and enthusiasm, the other side of the coin of “governation.”^4^

To give a glimpse of how challenging it was to create from scratch a novel kind of cryo-collection under such circumstances, I will draw on the issue of institutionalized ethics. With regard to cryo-A-cells as a clinical object, the main ethics-related question revolved around donor burden and its necessity. Cryo-A-cells as envisaged by X-Bank were meant to be harvested from donors along with fresh A-cells. Thus, most of the cells harvested were meant to be directly transplanted to a specific patient in need, while a small surplus amount would be cryopreserved. What is important to note here is that retrieving that surplus amount takes slightly longer than a usual procedure meant to extract a fresh transplant of sufficient size. As it was unclear whether the additional donor burden would ever result in an additional transplantation, one of the regulatory authorities in charge did not want to issue a manufacturing permit. Thus, the suspension of a clinical object in time and space, to render it available whenever (Lemke 2023b) and for whomever necessary, was at odds with the logics of biomedical ethics regulation. For a long time, attempts to reassure authorities that the cryo-A-cells project would generate a valuable medical resource were unsuccessful, because they did not address an important concern: Is it legitimate to extract and suspend living cells from a human body if it is entirely unclear whether they will ever be used? The open-endedness here is a different one than in the case of biological material preserved for research (Hoeyer 2008; Spencer et al 2012). What is unknown is not the exact purpose and trajectory of banked cellular life, but rather if there will be *any* purpose and trajectory beyond suspension itself.

As the next section shows in detail, cryobanking projects do not suspend biological material for it to be completely used up but to be accessible just in case it might be of use at some time in the future. Radical uncertainty with regard to future utilization is willingly tolerated and embraced in the regulation of research innovation (Brown 2003; Tutton 2011). When it comes to the approval of clinical objects, however, it is not: authorities are not willing to provide licenses for the manufacturing of medicinal products that may or may not be used. If such an approval is to be granted in the context of altruistic donation of somatic tissue, there is even less acceptance for the possibility of non-utilization. Given that this possibility is an integral characteristic of any project of suspension, however, X-Bank’s cryo-A-cells initiative was met with distrust by the inexorable regulation it was destined to deal with.

## Suspension at odds with politics of preparedness

The cryotechnological suspension of A-cells obviously runs counter to established ways of imagining and governing biomedical innovations. However, one might object that it was not meant to be part of business as usual but an intervention against the background of the looming risk of crises. After all, the idea of systematically cryopreserving A-cells was among other things framed as an apt response to the disruption of donation systems caused by the outbreak of the COVID-19 pandemic. At first glance, a stockpiling project like this does indeed fit into the political rationale of preparedness (Keck 2017) that gained new momentum in the early 2020s. Such a perfect match with timely concerns is also what the people who kicked it off had in mind. In a public relations piece published in several media outlets during the first wave of the pandemic in the Global North, for example, the cryo-A-cells project was presented as a matter of preparedness for which there had long been a need. It was framed as an investment in dealing with the current disruptions and potential crises of comparable scope that were yet to come. However, the slow and rigid response of regulatory authorities has ultimately turned the cryopreservation of cryo-A-cells into anything but a swift practice of addressing issues rendered visible by COVID-19 and preparing for future disruptions. This prompts the question of why the pandemic did not bring the expected enthusiasm and acceleration in regulation.

The A-cells to be cryopreserved in X-Bank’s facilities are meant to be movable to anywhere they are needed. Calling this stockpiling initiative into life draws on and affirms transnational flows of biomedical transplants. The project is supposed to become part of a global assemblage of cell donations (Brown and Williams 2015). Stockpiling, on the other hand, was and is mainly a matter of national or supra-national preparedness. As Andreas Folkers (2019) discusses in his genealogical account, the emergence and development of this practice and discourse are deeply imbricated with the nation state. From the centralization of power in early states, to the mercantile prohibition of hoarding, to the liberal framing of the latter as a market device, to military security policies: stockpiling has long remained something that is first and foremost organized and intervened in by the state (ibid.). When it comes to the biomedical and pharmaceutical domain in particular, stockpiling has been shown to be a way of facing the globalized circulation of disease threats from the vantage point of national security (Elbe et al 2014).^5^*Suspending in order to share*, as envisioned by X-Bank, contradicts these patterns. As an initiative called into life to boost cross-border flows of therapeutic material and increase the overall preparedness of the transnational cell gift economy, it is at odds with familiar preparedness regimes.

The collection of cryo-A-cells is not only too transnational but also too open-ended and inexhaustible to be governed as a stockpiling project. In making a distinction between stockpiling and storage, Frédéric Keck (2017) argues that an orientation toward sharing may well be the goal of cryopreservation initiatives. However, as an anthropologist with a long record of studying the social life of viruses, Keck aptly observes that such a detachment from property relations is incompatible with “the imagination of scarcity” (ibid., p. 137) and “emotions of panic and inequity” (ibid., p. 136) that are typically enacted in the context of a looming pandemic. Rather, he argues, freezing biological material in order to share it is characteristic of long-term open-ended collections designed to be gradually expanded to work in the best possible ways. Keck makes this case by distinguishing between stockpiling vaccines and antivirals on the one hand and storing virus samples on the other. The former are hoarded and cooled for a limited time frame as a short-term, perishable buffer for a scenario of material shortage. The latter, on the other hand, are ideally preserved at ultra-low temperatures to be readily made available forever, whenever and from wherever. Availability in this case does not equal rapid consumability but refers to the possibility of access. In the event of a public health crisis, such collections are not supposed to be used up. Instead, they are meant to be accessed as the largest possible pool in case a specific sample from the past might be useful for classifying a newly circulating virus. According to Keck, such and similar biobanking initiatives are capable of circumventing the “‘dirty politics’ of sovereignty and property” (ibid., p. 128) by rendering their cryo-collections legible and “easily accessible [as] digital information” (ibid., p. 127).

Biological material such as A-cells is also not accumulated to be consumed in total. It is first and foremost represented in virtual registries that allow for a quick identification of potentially useful specimens. Such collections are organized according to economies of scale: their value grows as they become larger (Brown and Williams 2015, p. 8). X-Bank’s cryo-A-cells initiative was designed in these very terms, as an accessible open-ended reserve; it did not mind ‘dirty politics’ and was supposed to render a cryo-collection useful for anyone at any time. With its promise of creating a buffer, it did certainly mimic politics of preparedness and logics of stockpiling; however, it was not exactly in line with them. It ran counter to the imagination of shortage that is constitutive of stockpiling projects as they are promoted, supported and funded in the context of pandemic preparedness. The absence of ‘dirty politics’ was thus a disadvantage: with no scarcity issue in the picture and no one to compete with in terms of access, there was a lack of an interested party.

Surprisingly, the cryopreservation of donated cells is not a singular practice; there is more than one logic of rendering such biological material storable. The idea of cryopreserving donated cells right after harvesting it from the donor and making it available for each and every transnational transplantation request is only one possible mode of preparing for crisis events. There are other options that fit well into familiar tropes of the politics of preparedness. The rare blood program of the Canadian Blood Services, for instance, includes a collection of cryopreserved erythrocytes to be shipped to Canadian hospitals in search of donated units of rare blood types. Its one and only goal is to “meet the needs of the highly diverse Canadian population” (Turner and Acker 2020, p. 1). Another, explicitly pandemic-born example is the US-based initiative to suspend bone marrow and peripheral blood. For some months in spring and summer 2020, all unrelated donor cell products to be transplanted to US patients had to be cryopreserved upon arrival and prior to transplantation; this obligation was reinstated as a recommendation against the backdrop of the rise of the Omicron variant in January 2022 (Koo et al 2022; Maurer et al 2022). While the strict cryo-only policy was soon reversed, allowing for fresh grafts to be transplanted again, the rates of cryopreservation remained high through the peak of the pandemic as compared to its almost complete absence in 2019 (NMDP n.d. b). In October 2020, the National Marrow Donor Program (NMDP) finally announced that it would offer cryopreservation services for all transplant centers in its network (PR Newswire 2020). Today, the corresponding program *Be The Match® BioBank* is well-established; it is based on cooperation with a public cord blood bank and a university-based cellular therapeutics center as the facilities in charge of cryopreservation and storage. It promises to “freeze product from any donor, domestic or international” (NMDP n.d. a) upon request of transplant centers. Thus, it aims at *suspending what has been shared,* which is a very different mode of preparing for crises than that envisaged by the cryo-A-cells project. It is a cryotechnological intervention that is meant to ensure that US transplant centers can access donated material intended for their patients even in times of infrastructural disruptions. As such, it is an initiative that is actually oriented toward national preparedness. What is more, it is not an open-ended collection of potentially useful specimens but an actual material buffer to be consumed. In other words, the US case is an example of how the cryotechnological suspension of cells can blend in with the governance of emergency in times of a pandemic if explicitly designed in line with what Keck calls its “‘dirty politics’ of sovereignty and property.”

## Discussion: outside politics or no politics?

The preceding sections have depicted two mismatches between X-Bank’s cryo-A-cells project and the political rationales that have hitherto framed and ordered the field in which it was developed. They vividly demonstrate that the project did not follow in the footsteps of those rationales but envisaged a hitherto unfamiliar pathway for intervention. To inquire into what prevents a smooth integration into the beaten tracks of politics, Stephenson and Papadopoulos (2006a; 2006b) have considered the question of “outside politics” with a focus on the open-endedness and idiosyncrasy of people’s experiences. They use the notion of outside politics to point out how certain experiences are ungraspable for political representation as they do not match with familiar ways of imagining and governing identities, biographies, and socialities. These authors argue that in and through their status of imperceptibility, such experiences carry the political potential of disrupting and resisting hegemonic practices of narrating and governing lives.

Even though Stephenson and Papadopoulos deal with the daily lives and circumstances of individuals, an important pillar of their argument can also be mobilized for the study of organized projects and practices—namely their interest in the political status of the singular and imperceptible. Like the spectrum of possible experiences, the spectrum of possible organized actions is broader than what established “bureaucratized and technocratic worlds and discourses” (Stephenson and Papadopoulos 2006a, p. 171) consist of. X-Bank’s cryo-A-cells project is a good example: in mobilizing the idea of suspension for the buffering of a clinical gift economy, it explored “unrealized trajectories, possibilities which do not yet exist” (ibid., p. 170). Instead of connecting to practices and discourses in familiar ways, it sought to forge new connections. As the preceding account shows, this very fact resulted in the cryo-A-cells initiative becoming neglectable and imperceptible for established political rationales—just like the excluded, un-represented and un-representable experiences discussed by Stephenson and Papadopoulos. However, the latter, Stephenson and Papadopoulos argue, gain political force from their marginalization; they can give rise to a distinct kind of politics itself, namely outside politics. The political illegibility of an organized project of suspension, on the other hand, did not grant it political potential. Its imperceptibility did not turn it into an “engine of socio-political transformation” (ibid., p. 138). Nor did the project succeed in inserting itself “into political disputes in [its] own terms” (ibid., p. 157).

Viewing the cryo-A-cells endeavor through the lens of the notion of outside politics underlines that it did not succeed in *finding* its own terms in the first place. A project of suspension positioned outside familiar political idioms and regimes was not “politically effective” (ibid., p. 158) at all. It neither contested nor resisted, but became in fact a “victim of exclusion” (ibid., p. 138). This insight prompts the conclusion that the politics of suspension is at best a conditional kind of politics that loses its potential once it fails to connect to other, familiar, politics in predictable ways. It might also be worthwhile to reconsider if there is a distinctive kind of politics around suspension at all, or if suspension is rather an idea and a practice that may or may not be successfully incorporated into some existing forms of politics.

## Conclusions

Biotechnologies have for a long time been viewed and analyzed as political vehicles—vehicles through which lives are governed. Cryotechnologies in particular are considered political because they facilitate unique ways of governing lives by suspending biological material in time and space. Hence, this article comes from a rather unusual place: it starts off from a particular project of cryotechnological suspension that was supposed to and yet failed to gain such political traction. My purpose was to understand its struggles to be acknowledged and realized by reconsidering the politics of suspension as conditional rather than distinctive.

To place the case study of a long envisaged, yet still not operational cryo-collection of donated cryo-A-cells in a broader context, I reviewed STS literature on similarly ill-fated biobanking endeavors. In juxtaposing such research findings with the cases cited in recent theoretical accounts on the rationale of suspension, I drew attention to the fact that the latter were supplements and extensions of established political rationales. To show that this is not always the case, I then took a granular look at the ways in which the cryo-A-cells project was at odds with familiar politics significant in its field. Finally, the notion of “outside politics” as a heuristic device allowed me to make sense of the lack of any transformative or resistant political potential that came with the mismatched project of suspension I studied. It confirmed the hypothesis derived from the selective review of biobanking studies: politics only discernably emerge around the idea of suspension once it is well integrated into other political regimes.

Obviously, no politics ever stands alone and unfolds independently from others. However, as the summarizing account of ill-fated biobanking initiatives and the detailed insights into the cryo-A-cells project as a mismatch indicate, the politics of suspension are not just interlinked with other politics to a greater or lesser extent. They do not even *emerge* as such if the idea of suspension is not explicitly mobilized to bring other, already established, ideas to perfection. Projects of suspension that seek to make new connections rather than to connect to well-known political idioms and norms seem doomed to fail to make any difference. Analyzing one ill-fated trajectory of this idea prompts the important conclusion that the power to arrest life as it comes with cryotechnologies is much more likely to unravel as such in entrenched and hegemonic constellations rather than to carry any transformative or disruptive potential.

This insight does not make the investigation of such cases less important. The significance of the discourse and practice of suspension lies precisely in its potential to align with and become part of other politics. Whether it is the politics of security in the agri-food sector, eco-modernist imaginaries of human–environment relations, or the neoliberal governance of reproduction and fertility—not unlike a chameleon, suspension as a vision and a technology successfully blends in with several contexts of governing life. Framing suspension as an idea that gives rise to a distinct sort of politics distracts from and disguises this political plasticity. It runs the risk of overestimating the role of a politics of suspension as compared to the politics that fostered their emergence in the first place. Hence, I suggest pursuing the research agenda around the idea of suspension in ways that explicitly acknowledge the conditionality of the corresponding politics. This implies two things. First, there is a need for empirical and genealogical insights into what it is that paves the way for technologies of suspension to be integrated into particular existing discourses and power formations. In the spirit of historical and genealogical work such as that presented by Joanna Radin (2017), Keck (2017) or Folkers (2019), we need to ask: Under what epistemic and material circumstances and for the sake of what expectations is suspension mobilized in the name of particular political ideas? Second, more emphasis needs to be placed on the ways in which the idea of suspension fails to make a difference or is even rejected. Accounts such as the one presented here are important, as they render tangible how and why certain interventions by means of suspension fail to align and become inoperable. Like Argudo-Portal and Domènech’s (2022) study, they can also demonstrate what alternatives are mobilized when the idea of suspension is dismissed, and what consequences they may entail. Overall, the investigation of cases of suspended suspension prevents such unrealized initiatives from falling into oblivion. By showing that the idea of suspension is not doomed to serve objectionable purposes and can assume form in projects beyond solidified “aggregates of governance,” STS scholarship will contribute to its open-endedness and diversification.

## Data Availability

The data that support the findings of this study are not publicly available due to privacy and ethical restrictions.

## Footnotes

1 In his concluding contribution to the panel “Storage: Infrastructures, Politics, Imaginaries” at the 2nd Joint 4S/ESOCITE Meeting in Cholula, México, Stefan Helmreich (2022) also used the notion of “buffering” to characterize how storage practices compensate for, defer, and absorb infrastructural disruptions. In doing so, he, among other things, referred to the paper on urban storage infrastructures previously presented by Moritz Kasper and recently published by the latter and Sophie Schramm (2023).

2 From time to time, some cryobanks are reportedly affected by disruptions of electricity or liquid nitrogen supply (Tomlinson 2018). However, most medium- and large-sized cryobanks such as X-Bank nowadays have well-maintained alarm and monitoring systems for both liquid nitrogen level and temperature as well as power loss back-up systems. The latter can compensate for several hours of power outage.

3 A purpose-oriented alternative to open-ended suspension, Argudo-Portal and Domènech (2022, 341) argue, may well have negative effects as the need to prove timely utilization is likely to favor certain (above all well-funded) research actors over others.

4 Over the last few years, X-Bank has also become involved in other projects and collaborations explicitly designed to resonate with the regional biotech innovation strategy. This further underscores the significance of aligning with “governation” to ensure economic and political viability.

5 While there has been a “new conceptualization of the problem of global health” (Lakoff 2017, 66) since the 2000s, international efforts in public health preparedness first and foremost place importance on surveillance and humanitarian initiatives (ibid., 71–74). Stockpiling has so far remained something to be initiated by and expected from national governments (Lakoff 2008, 2020; Mezes and Opitz 2020).
